# Can NAD(P)^+^ transhydrogenase (NNT) mediate a physiologically meaningful increase in energy expenditure by mitochondria during H_2_O_2_ removal?

**DOI:** 10.1016/j.jbc.2021.100377

**Published:** 2021-03-08

**Authors:** Tiago R. Figueira, Annelise Francisco, Jason R. Treberg, Roger F. Castilho

**Affiliations:** 1 School of Physical Education and Sport of Ribeirão Preto, University of São Paulo (USP), Ribeirão Preto, Brazil; 2 Department of Pathology, Faculty of Medical Sciences, University of Campinas (UNICAMP), Campinas, Brazil; 3 Department of Biological Sciences, University of Manitoba, Winnipeg, Manitoba, Canada; 4 Centre on Aging, University of Manitoba, Winnipeg, Manitoba, Canada

Smith *et al*. (1) studied skeletal muscle mitochondria (SMM) from different inbred mouse strains (C57BL/6NJ versus C57BL/6J; NNT function is absent in the latter) and concluded that NNT activity mediates meaningful increases in respiration when NADPH-dependent mitochondrial H_2_O_2_ removal is stimulated. To be valid, this contention requires the NNT reaction stoichiometry to be ∼200 H^+^ translocated per NAD(P) hydride transferred based on expected stoichiometries in mitochondrial respiration (at least 6H^+^:1O) and some of the authors’ reported values (1). The authors argued that NNT’s stoichiometry may be altered when the enzyme is generating NADPH (1), but a ratio of 200 H^+^ per hydride transfer challenges recent advances on the structure of NNT (2, 3), which provide strong evidence these two molecular events are tightly coupled with a ratio of 1:1. Smith *et al*. (1) assert NADPH-dependent H_2_O_2_ removal can be linked to respiration by adding auranofin plus bis-chloroethylnitrosourea to mitochondria. However, these compounds caused similar decreases (∼35%, see Fig. 4D (1)) in O_2_ consumption in both mouse strains, indicating suppression of respiration irrespective of NNT flux and making conclusions based on these inhibitors ambiguous. Finally, previous studies comparing mitochondria from mice with or without functional NNT (using genetically closer controls) found no evidence of NNT-mediated flux sufficient to be measured as increased respiration (4, 5). While the findings of Smith *et al*. (1) are attractive, we contend more direct evidence is needed before the role of redox circuits through NNT can be definitively understood in the context of energy expenditure.

## Conflict of interest

The authors declare that they have no conflicts of interest with the contents of this article.
